# Potential Mechanism and Effects of Different Selenium Sources and Different Effective Microorganism Supplementation Levels on Growth Performance, Meat Quality, and Muscle Fiber Characteristics of Three-Yellow Chickens

**DOI:** 10.3389/fnut.2022.869540

**Published:** 2022-04-15

**Authors:** Junjing Xue, Chengkun Fang, Rui Mu, Ruiwen Zhuo, Yuanyuan Xiao, Yiqing Qing, Jiaxi Tang, Rejun Fang

**Affiliations:** ^1^College of Animal Science and Technology, Hunan Agricultural University, Changsha, China; ^2^Hunan Co-Innovation Center of Animal Production Safety, Changsha, China

**Keywords:** selenium yeast, effective microorganism, growth performance, meat quality, muscle fiber characteristic

## Abstract

A trial was conducted to investigate the effects of different Se sources, including sodium selenite (S-Se) and selenium yeast (Y-Se) and different effective microorganism (EM) addition levels on growth performance, meat quality, and muscle fiber characteristics of three-yellow chickens and its potential mechanism. A total of 400 birds were randomly distributed into 4 groups (S-Se, S-Se + EM, Y-Se, and Y-Se + EM groups) consisting of a 2 × 2 factorial arrangement. The main factors were the source of Se (I_Se_ = inorganic Se: 0.2 mg/kg S-Se; O_Se_ = organic Se: 0.2 mg/kg Y-Se) and the level of EM (H_EMB_ = high EM: 0.5% EM; Z_EMB_ = low EM: 0% EM). Each treatment had 5 replicates and each replicate consisted of 20 broiler chickens. The trial lasted for 70 days. The results showed that, in breast muscle, the broiler chickens fed O_Se_ source decreased the pH_24h_, drip loss, shear force, perimeter, cross-sectional area, and diameter, but increased the ${\text{a}}_{24\text{h}}^{*}$ and density compared with the broiler chickens fed I_Se_ source (*p* < 0.05); broiler chickens supplied with H_EMB_ level decreased the cross-sectional area and diameter, but increased the pH_24h_, ${\text{a}}_{24\text{h},}^{*}$ and density compared with the broiler chickens supplied with Z_EMB_ level (*p* < 0.05). In thigh muscle, O_Se_ source and H_EMB_ level also could improve the meat quality and change muscle fiber characteristics of broiler chickens (*p* < 0.05). Meat quality was correlated with the muscle fiber characteristics (*p* < 0.05). O_Se_ source and H_EMB_ level could regulate the expression levels of muscle fiber-relative genes in the breast and thigh muscles (*p* < 0.05). In conclusion, O_Se_ source and H_EMB_ level could improve the meat quality of the breast and thigh muscles of three-yellow chickens by changing the muscle fiber characteristics, and they changed the muscle fiber characteristics by regulating the expression levels of muscle fiber-relative genes.

## Introduction

Three-yellow chicken is one of the most famous indigenous breeds in China. They have a high content of linolenic acid and amino acid, and a low content of sinapic acid in the meat. As people's living standard has improved, the need for high-quality meat products has greatly increased (1). However, large-scale feeding of three-yellow chicken leads to a decrease in meat quality. Thus, more research has gravitated toward improving the meat quality of broiler chicken (2–4).

Sodium selenite (S-Se), selenium yeast (Y-Se), selenomethionine, and nano-selenium can be supplied in animal feed (5). Se is an essential trace element and a key part of glutathione peroxidase. Compared with S-Se, Y-Se has greater effects and is widely used (6). Dietary Y-Se supplementation can improve the carcass quality, antioxidant status, nutrition digestibility, Se deposition, and meat quality of Cobb 500 broilers, laying hen, and sheep (4, 7, 8). Probiotics such as *Lactobacillus*, yeast, and *Bacillus subtilis* as the possible substitute for antibiotics have attracted a lot of attention (2, 9). Previous studies had shown that, when supplied with *Lactobacillus*, it could improve the nutrient absorption, intestinal integrity, meat quality, and keep the balance of gut microbiota of weaning piglets, ducks, and Arbor Acres (AA) broilers (3, 10–12). Dietary yeast can improve the antioxidant status and meat quality of AA and Ross broilers (2, 10, 13). Effective microorganism (EM) mainly included *Lactobacillus* and yeast in this experiment. However, the effect of Y-Se and EM on the growth performance, meat quality, and muscle fiber characteristics of three-yellow chickens is still unknown.

Meat quality is reflected by many indexes such as pH value, color, flavor, drip loss, cooking loss, and shear force. The level of glycolysis and the content of lactic acid and oxidation level in the muscle can influence the meat quality (14–17). Previous studies had shown that, when supplied with Y-Se, *Lactobacillus* and yeast could increase the antioxidant capacity of ducks, aging mice, Ross broilers, and pigs by increasing the activities of glutathione peroxidase and superoxide dismutase and decreasing the contents of thiobarbituric acid reactive substances and malondialdehyde (2, 13, 18–20). The increase in antioxidant capacity prevents the oxidation of myoglobin, which can deepen the muscle color (21, 22), and it prevents the oxidation of muscle and keeps the cytomembrane integrity, thus decreasing the L^*^, drip loss, and cooking loss (14, 16, 23). Previous studies had found a correlation between meat quality and muscle fiber characteristics (24–26). However, there is less information about this correlation in broiler chickens. In this experiment, we investigated the effects of different Se sources and different EM addition levels on the growth performance, meat quality, and muscle fiber characteristics of three-yellow chickens, and studied the correlation between meat quality and muscle fiber characteristics, and then illustrated the underlying mechanism.

## Materials and Methods

### Animals and Diets

A total of 400 one-day-old healthy three-yellow chickens (male, average initial weight was 35.72 ± 0.05 g) were randomly distributed into 4 groups (S-Se, S-Se + EM, Y-Se, and Y-Se + EM groups) consisting of a 2 × 2 factorial arrangement. The main factors were the source of Se (I_Se_ = inorganic Se: 0.2 mg/kg S-Se; O_Se_ = organic Se: 0.2 mg/kg Y-Se) and the level of EM (H_EMB_ = high EM: 0.5% EM; Z_EMB_ = low EM: 0% EM). The basal diet met the nutrient requirements (except for microelement) of broiler chickens according to NRC 1994 and NY/T33 2004 (Table 1). The additional level of microelement was determined according to our previous study. The broiler chickens in the S-Se and S-Se + EM groups were fed the basal diet supplemented with 0.2 mg/kg S-Se, and in the Y-Se and Y-Se + EM groups were fed the basal diet supplemented with 0.2 mg/kg Y-Se. Meanwhile, the S-Se + EM and Y-Se + EM groups were fed the basal diet supplemented with 0.5% EM (Bokaxi, Changsha, China) which mainly included *Lactobacillus* and yeast at a dose of 2 × 10^8^ cfu/kg and 3 × 10^7^ cfu/kg, respectively. The analyzed value of Se in the diets is shown in Table 2. The trial lasted for 70 days. The birds had *ad libitum* access to food and water. Each treatment had 5 replicates, and each replicate consisted of 20 broiler chickens. Each replicate was raised in a single cage having the dimensions 160×160×100 cm (length × width × height). The room temperature was maintained constant at 22 ± 1°C. The relative humidity was maintained at 55 ± 5%. The lighting schedule was 23 h light:1 h dark. Birds were weighed in the morning at 1, 35, and 70 days, and feed intake was recorded each day. At the end of the trial, body weight (BW), average daily feed intake (ADFI), average daily gain (ADG), and feed conversion ratio (FCR) were calculated.

**Table 1:** Composition of the basal diet and nutrition level (dry matter).

**Ingredient**	**Content, %**		**Nutrient level^**c**^**	**Content**	
	**1 to 35 d**	**36 to 70 d**		**1 to 35 d**	**36 to 70 d**
Corn	59.00	65.00	DE (Mcal/kg)	3.00	3.12
Soybean meal	30.28	20.00	Crude protein (%)	21.26	16.32
Cottonseed meal	2.00	4.50	Crude lipid (%)	4.35	4.56
Fish meal	3.20	-	Crude ash (%)	6.08	5.93
Wheat bran	-	3.15	Ca (%)	0.97	0.88
Soybean oil	1.45	3.00	Total P	0.72	0.65
Ca(H_2_PO_4_)_2_	1.05	1.10	Available P (%)	0.46	0.36
Limestone	1.50	1.70	Lys (%)	1.11	0.86
Choline chloride	0.10	0.10	Met (%)	0.53	0.41
DL–Met	0.18	0.15	Met + Cys (%)	0.86	0.69
NaCl	0.24	0.20	Thr	0.80	0.69
NaHCO3	-	0.10			
Mineral premix^a^	0.98	0.98			
Vitamin premix^b^	0.02	0.02			
Total	100.00	100.00			

a *The mineral premix provided per kilogram of complete feed: Cu, 1.06 mg; Fe, 84.67 mg; Zn, 29.24 mg; Mn, 4.43 mg.*

b *The vitamin premix provided per kilogram of complete feed: vitamin A, 6,500 IU; vitamin D_3_, 2,000 IU; vitamin E, 16 IU; vitamin K_3_, 2 mg; vitamin B_1_, 2 mg; vitamin B_2_, 5 mg; vitamin B_6_, 1.6 mg; vitamin B_12_, 0.015 mg; D–biotin, 0.12 mg; D–pantothenic acid, 10 mg; folic acid, 1 mg; nicotinamide, 20 mg.*

c *The crude protein, crude lipid, and crude ash were measured values, and the others were calculated values*.

**Table 2:** Concentration of Se in experimental diets (mg/kg).

**Treatments**	**Supplemented values**	**Analyzed value**	
	**1 to 70 d**	**1 to 35 d**	**36 to 70 d**
S–Se	0.200	0.215	0.210
S–Se + EM	0.200	0.220	0.215
Y–Se	0.200	0.215	0.217
Y–Se + EM	0.200	0.209	0.213
SEM	0.002	0.002	
*P* value	0.592	0.593	

*S-Se, sodium selenite; S-Se + EM, sodium selenite + 0.5% EM; Y-Se, selenium yeast; Y-Se + EM, selenium yeast + 0.5% EM*.

### Sample Collection

At the end of the experiment, birds were fasted for 24 h before slaughter, and electrically stunned and euthanized by cervical dislocation. The right breast and thigh muscles were collected from one bird in each replicate. Some of the muscles were fixed in 4% formalin for morphometric analyses, some were used for meat quality examination, and some were immediately frozen at −80°C for quantitative real-time polymerase chain reaction (qRT-PCR) analysis.

### Meat Quality

Meat quality of the right breast and thigh muscles was determined according to the method developed by Meng et al. with a slight modification (27). The pH values were measured at 45 min, 24 h, and 48 h postmortem using a portable pH meter (Mettler-Toledo, Shanghai, China). At the same time, meat colors, which included L^*^, a^*^, and b^*^, were determined by Minolta chroma meter (Konica Minolta, Tokyo, Japan). During 45 min after postmortem, the surface water of muscles (around 30 g) was removed, and the initial weight was recorded. After hanging vertically at 4°C for 24 h, the final weight was recorded. The drip loss was calculated as follows:


(1) $$\text{Drip loss }\left(\%\right)=\frac{\text{initial weight }-\text{ final weight}}{\text{initial weight}}\times 100\;$$

The muscles were trimmed to 2 cm × 2 cm × 1 cm, and the initial weight was recorded. The meats were packed by bag and immersed in 80°C water until the internal temperature of meats reached 74°C. The meats were cooled and the final weight was recorded after removing the surface water of meats. The cooking loss was calculated as follows:


(2) $$\text{Cooking loss }\left(\%\right)=\frac{\text{initial weight }-\text{ final weight}}{\text{initial weight}}\times 100\;$$

After measuring the cooking loss, the shear force was measured using digital muscle shear apparatus (Stable Micro Systems, Surrey, UK).

### Muscle Fiber Characteristics

The right breast and thigh muscles were embedded in paraffin and cut into 10 μm sections (horizontal axis). The samples were dehydrated *via* a series of incubations in xylene and ethanol solutions, and then stained with hematoxylin and eosin (HE staining), and observed through a microscope (400×) (Nikon, Tokyo, Japan). Three areas (each area was 0.15 mm^2^) in a slide (6 slides in each treatment) were randomly selected to examine the perimeter, cross-sectional area, diameter, and density of muscle fiber using CaseViewer.

### qRT-PCR Analysis

The relative expression levels of myogenic factor 5 (*Myf5*), myogenin (*MyoG*), myogenic regulatory factor 4 (*MRF4*), calpain 2 (*CAPN2*), calpain 3 (*CAPN3*), calpastatin (*CAST*), myostatin (*MSTN*), myocyte enhancer factor 2A (*MEF2A*), myocyte enhancer factor 2D (*MEF2D*), fast myosin heavy chain (*FM*), slow myosin heavy chain (*SM*), insulin-like growth factor I (*IGF-I*), and insulin-like growth factor II (*IGF-II*) in the right breast and thigh muscles were detected by qRT-PCR. The primers (Sangon Biotech, Shanghai, China) used are listed in Table 3. The β*-actin* gene was chosen as the reference gene for sample normalization. qRT-PCR reactions were carried out in a –Bio-Rad CFX96 touch qPCR system (Applied Biosystems, Foster City, USA) in 20 μl volumes that contained the following components: 10 μl of SYBR Green Mix (Takara, Changsha, China), 2 μl cDNA (1,000 ng/μl), 0.4 μl of each primer (10 mM), and 7.2 μl dH_2_O, followed by 40 cycles of 95°C for 30 s, 55°C−58°C for 30 s, and 72°C for 30 s. Finally, a melt curve analysis was used to detect the single product. The relative expression level was analyzed using the 2^−ΔΔCT^ method. The amplification efficiencies of all primes ranged from 0.90 to 1.00. All samples were tested in triplicate.

**Table 3:** The primers for qRT-PCR.

**Genes**	**Primer Sequence (5' to 3')**	**Accession number**	**Size (bp)**
*Myf5*	F: CAACCAGAGACTCCCCAAA	NM_001030363.1	87
	R: TCCACCTGTTCCCTCAAGA		
*MyoG*	F: GCAGAGGTTTTACGATGGGG	NM_204184.1	102
	R: CTTTCAGGGCACAGGGTCAC		
*MRF4*	F: AAAAGGCGGACTGTGGC	D10599.1	234
	R: GGAATGGTCGGAAGCG		
*CAPN2*	F: ACCACTGAAGGCTTTGAGG	NM_205080.1	264
	R: GGGATTTCGGATTCTGATG		
*CAPN3*	F: TTGCTTGCCTGACACTGAAT	NM_001004405.2	162
	R: GCTGGTTGTTGTATGTGGGTAG		
*CAST*	F: GGATGAATCAGCACTGGACA	NM_001137650.1	149
	R: GGAGGCTACCTTCTCGTTTT		
*MSTN*	F: GGCTCTGGATGGCAGTAGTC	DQ912835.1	293
	R: TAATCGTCTCGGTTGTGGC		
*MEF2A*	F: TCACGAGAATAATGGACGAAC	NM_204864.3	158
	R: AGTGCTGGCATACTGAAAGAG		
*MEF2D*	F: CTTGATGAAGAAAGCCTACGAG	NM_001031600.3	173
	R: TGATGTCCGCATTGGTCC		
*FM*	F: CTGTGCTATCCTCGTGTCAAG	AF272033.1	202
	R: CAAATCCAGCGATGTCCA		
*SM*	F: GCTCCACTCGCAGAACAC	U85022.1	89
	R: CAGCATCCTCCACCTCG		
*IGF - I*	F: GGTTGATGCTCTTCAGTTCGTA	M32791.1	96
	R: CCCTTGTGGTGTAAGCGTCT		
*IGF - II*	F: GGGACAGGGGCTTCTACTTC	NM_001030342.3	142
	R: CGCTCTGACTTGACGGACTT		
*B - actin*	F: TGAAGCCCAGAGCAAAAGA	L08165.1	275
	R: TACGACCAGAGGCATACAGG		

*Myf5, myogenic factor 5; MyoG, myogenin; MRF4, myogenic regulatory factor 4; CAPN2, calpain 2; CAPN3, calpain 3; CAST, calpastatin; MSTN, myostatin; MEF2A, myocyte enhancer factor 2A; MEF2D, myocyte enhancer factor 2D; FM, fast myosin heavy chain; SM, slow myosin heavy chain; IGF-I, insulin-like growth factor I; IGF - II, insulin - like growth factor II; β-actin, beta-actin*.

### Statistical Analysis

The experimental design was a 2 × 2 factorial arrangement, and the main factors were the source of Se and the level of EM. Data were analyzed by –two-way ANOVA using SPSS 22.0 (SPSS. Inc., Chicago, USA), which included the main effects of Se source, EM level, and their interaction (Se source × EM level). Tukey's multiple-range test was used to analyze the differences. All data were further subjected to one-way ANOVA. When overall differences were significant, the differences were tested by Duncan's multiple-range test (SPSS 22.0). The correlations between the meat quality and muscle fiber characteristics in the breast and thigh muscles were analyzed by SPSS 22.0. The level of significance was set at *p* < 0.05, and high level of significance was set at *p* < 0.01. The results are presented as the mean values and SEM.

## Results

### Growth Performance

The effect of Se source and EM level on growth performance is shown in Table 4. Se source influenced FCR of three-yellow chickens (*p* < 0.05). There was no interaction between Se source and EM level regarding growth performance (*p* > 0.05). Broiler chickens fed O_Se_ source decreased the FCR during 1–35 days compared with the broiler chickens fed I_Se_ source (*p* < 0.05). During 1–35 days, a greater FCR was observed in the S-Se + EM group compared with the Y-Se and Y-Se + EM groups (*p* < 0.05), whereas there were no significant differences among the S-Se, Y–Se, and Y-Se + EM groups, or between the S-Se and S-Se + EM groups (*p* > 0.05).

**Table 4:** Effects of different Se sources and different bacteria supplementation levels on the growth performance of three-yellow chickens.

**Items**	**Groups**				**Main effect of S**		**Main effect of E**		**SEM**	***P-*** **value**			
	**S – Se**	**S – Se + EM**	**Y – Se**	**Y – Se + EM**	**I_**Se**_**	**O_**Se**_**	**Z_**EMB**_**	**H_**EMB**_**		**Treatment**	**S**	**L**	**S × L**
BW (g)													
Initial	35.80	35.65	35.76	35.68	35.73	35.72	35.78	35.67	0.05	0.735	0.963	0.294	0.746
35 d	714.05	702.63	716.21	725.82	708.34	721.02	715.13	714.23	4.12	0.272	0.132	0.911	0.206
70 d	1,909.01	1,912.58	1,898.09	1,883.30	1,910.80	1,890.69	1,903.55	1,897.94	11.10	0.815	0.405	0.814	0.701
ADG (g)													
1 to 35 d	19.23	18.96	19.43	19.68	19.09	19.56	19.33	19.32	0.12	0.171	0.055	0.972	0.256
36 to 70 d	34.14	34.46	33.26	33.07	34.30	33.16	33.70	33.76	0.32	0.375	0.095	0.920	0.697
1 to 70 d	26.61	26.65	26.30	26.36	26.63	26.33	26.45	26.51	0.16	0.843	0.386	0.873	0.970
ADFI (g)													
1 to 35 d	36.78	37.19	36.69	37.32	36.99	37.00	36.74	37.25	0.23	0.759	0.970	0.300	0.830
36 to 70 d	98.82	97.97	96.79	96.40	98.40	96.60	97.81	97.19	0.69	0.620	0.223	0.668	0.875
1 to 70 d	67.50	67.36	66.55	66.80	67.43	66.67	67.02	67.08	0.39	0.832	0.381	0.946	0.816
FCR (g:g)													
1 to 35 d	1.92^ab^	1.94^a^	1.89^b^	1.89^b^	1.93*	1.89	1.90	1.91	0.01	0.011	0.002	0.284	0.198
36 to 70 d	2.89	2.84	2.91	2.92	2.87	2.92	2.90	2.88	0.02	0.650	0.324	0.664	0.516
1 to 70 d	2.54	2.53	2.53	2.54	2.53	2.54	2.54	2.53	0.01	0.994	0.884	0.942	0.826

*S-Se, sodium selenite; S-Se + EM, sodium selenite + 0.5% EM; Y-Se, selenium yeast; Y-Se + EM, selenium yeast + 0.5% EM. S, Se source; E, EM level; S × E, interaction between Se source and EM level; I_Se_, inorganic Se; O_Se_, organic Se; H_EMB_, high EM; Z_EMB_, low EM; BW, body weight; ADG, average daily gain; ADFI, average daily feed intake; FCR, feed conversion ratio.*

a, b *Values of group in the same row with the same superscript or absence of a superscript were not significantly different (p > 0.05).*

* *Values of the main effect of S in the same row were significantly different (p <0.05)*.

### Meat Quality in the Breast and Thigh Muscles

The effect of Se source and EM level on meat quality in the breast and thigh muscles is shown in Table 5. In breast muscle, the present experiment showed significant interactions between the Se source and EM level regarding pH_24h_ and drip loss (*p* < 0.05). Compared with the broiler chickens fed I_Se_ source, broiler chickens fed O_Se_ source decreased the pH_24h_, drip loss, and shear force, and increased the ${\text{a}}_{24\text{h}}^{*}$ (*p* < 0.05). Broiler chickens supplied with H_EMB_ level increased pH_24h_ and ${\text{a}}_{24\text{h}}^{*}$ compared with the broiler chickens supplied with Z_EMB_ level (*p* < 0.05). In detail, the S-Se + EM group had a greater pH_24h_ compared with the other groups, and the S-Se group had a greater pH_24h_ compared with the Y-Se + EM group (*p* < 0.05). The Y-Se + EM group had a greater ${\text{a}}_{24\text{h}}^{*}$ compared with the other groups (*p* < 0.05). The drip loss in the Y-Se + EM group was lower than that in the S-Se + EM and Y-Se groups (*p* < 0.05). Shear force in the Y-Se + EM group was lower than that in the S-Se and S-Se + EM groups (*p* < 0.05).

**Table 5:** Effects of different Se sources and different bacteria supplementation levels on the meat quality of three-yellow chickens.

**Items**	**Groups**				**Main effect of S**		**Main effect of E**		**SEM**	***P-*** **value**			
	**S – Se**	**S – Se + EM**	**Y – Se**	**Y – Se + EM**	**I_**Se**_**	**O_**Se**_**	**Z_**EMB**_**	**H_**EMB**_**		**Treatment**	**S**	**E**	**S × E**
Breast muscle													
pH_45min_	5.86	5.62	5.75	5.72	5.74	5.74	5.81	5.67	0.05	0.462	0.954	0.201	0.351
pH_24h_	5.70^b^	6.01^a^	5.62^bc^	5.59^c^	5.85*	5.60	5.66	5.80^§^	0.04	0.000	0.000	0.001	0.000
pH_48h_	5.79	5.86	5.81	5.81	5.83	5.81	5.80	5.83	0.03	0.903	0.827	0.641	0.598
${\text{a}}_{45\text{min}}^{*}$	1.59	1.74	2.12	1.85	1.66	1.98	1.85	1.80	0.11	0.382	0.154	0.789	0.349
${\text{b}}_{45\text{min}}^{*}$	6.78	6.86	5.43	6.26	6.82	5.80	6.03	6.59	0.31	0.333	0.135	0.472	0.560
${\text{L}}_{45\text{min}}^{*}$	42.05	45.04	41.36	43.60	43.55	42.48	41.67	44.24	0.74	0.330	0.476	0.095	0.802
${\text{a}}_{24\text{h}}^{*}$	1.40^b^	1.54^b^	1.71^b^	3.43^a^	1.50	2.40*	1.58	2.08^§^	0.26	0.021	0.016	0.033	0.062
${\text{b}}_{24\text{h}}^{*}$	8.51	8.21	8.76	8.16	8.33	8.46	8.63	8.19	0.21	0.817	0.854	0.408	0.767
${\text{L}}_{24\text{h}}^{*}$	48.30	47.10	47.80	45.07	47.79	46.58	48.02	45.94	0.63	0.287	0.321	0.135	0.542
${\text{a}}_{48\text{h}}^{*}$	2.09	1.16	1.51	2.07	1.72	1.83	1.80	1.77	0.19	0.332	0.675	0.638	0.085
b*_48h_	7.8	8.23	9.61	8.92	8.04	9.26	8.71	8.53	0.34	0.289	0.082	0.843	0.412
${\text{L}}_{48\text{h}}^{*}$	42.27	43.86	45.59	42.87	43.07	44.50	43.93	43.46	0.73	0.419	0.452	0.708	0.183
Drip loss (%)	2.74^bc^	3.75^a^	3.14^b^	2.34^c^	3.14*	2.74	2.94	2.90	0.17	0.006	0.026	0.573	0.002
Cooking loss (%)	20.37	20.94	20.90	20.18	20.73	20.54	20.70	20.56	0.37	0.870	0.890	0.930	0.450
Shear force (N)	12.15^a^	12.9^a^	10.32^ab^	8.35^b^	12.47*	9.48	11.24	10.62	0.60	0.023	0.004	0.498	0.151
Thigh muscle													
pH_45min_	5.98	5.95	5.99	5.97	5.97	5.98	5.99	5.96	0.03	0.954	0.810	0.620	0.936
pH_24h_	6.30	6.22	6.25	6.28	6.26	6.27	6.27	6.25	0.02	0.688	0.902	0.652	0.276
pH_48h_	6.44	6.49	6.41	6.46	6.47	6.44	6.42	6.48	0.03	0.816	0.650	0.408	0.988
${\text{a}}_{45\text{min}}^{*}$	6.13	5.04	6.34	6.63	5.58	6.46	6.23	5.72	0.30	0.286	0.152	0.507	0.261
${\text{b}}_{45\text{min}}^{*}$	5.79	6.53	5.19	6.17	6.16	5.56	5.36	6.31^§^	0.21	0.085	0.224	0.047	0.747
${\text{L}}_{45\text{min}}^{*}$	40.6	42.64	39.36	40.05	41.62	39.67	39.91	41.35	0.51	0.113	0.057	0.158	0.474
${\text{a}}_{24\text{h}}^{*}$	6.04^c^	11.09^b^	6.56^c^	13.96^a^	8.92	10.26	6.30	12.32^§^	0.99	0.000	0.072	0.000	0.193
${\text{b}}_{24\text{h}}^{*}$	6.57^b^	4.34^c^	4.66^c^	7.72^a^	5.62	5.97*	5.62	6.03	0.40	0.000	0.046	0.227	0.000
${\text{L}}_{24\text{h}}^{*}$	40.22	42.48	39.82	38.69	41.35*	39.34	39.99	40.58	0.53	0.074	0.037	0.531	0.080
${\text{a}}_{48\text{h}}^{*}$	5.93^b^	3.73^c^	5.29^bc^	10.69^a^	4.67	7.60*	5.56	6.71^§^	0.75	0.000	0.001	0.032	0.000
${\text{b}}_{48\text{h}}^{*}$	5.61^ab^	3.83^b^	4.57^b^	6.80^a^	4.85	5.52	5.09	5.32	0.38	0.022	0.110	0.687	0.004
${\text{L}}_{48\text{h}}^{*}$	41.54^a^	42.64^a^	36.40^b^	35.00^b^	42.17*	35.70	38.97	39.36	1.17	0.018	0.003	0.926	0.456
Drip loss (%)	2.99^b^	4.22^a^	2.99^b^	2.27^b^	3.48*	2.63	2.99	2.92	0.21	0.003	0.003	0.306	0.003
Cooking loss (%)	17.07	15.43	15.41	15.68	16.37	15.53	16.15	15.58	1.05	0.948	0.770	0.780	0.690
Shear force (N)	12.39^a^	10.21^ab^	8.45^b^	8.75^b^	11.30*	8.62	10.42	9.37	0.58	0.039	0.012	0.301	0.183

*S-Se, sodium selenite; S-Se + EM, sodium selenite + 0.5% EM; Y-Se, selenium yeast; Y-Se + EM, selenium yeast + 0.5% EM. S, Se source; E, EM level; S × E, interaction between Se source and EM level; I_Se_, inorganic Se; O_Se_, organic Se; H_EMB_, high EM; Z_EMB_, low EM.*

a−c *Values of group in the same row with the same superscript or absence of a superscript were not significantly different (p > 0.05).*

* *Values of the main effect of S in the same row were significantly different (p < 0.05).*

§ *Values of the main effect of E in the same row were significantly different (p < 0.05)*.

In the thigh muscle, Se source and EM level showed significant interactions on ${\text{b}}_{24\text{h}}^{*}$, ${\text{a}}_{48\text{h}}^{*}$, ${\text{b}}_{48\text{h}}^{*}$, and drip loss (*p* < 0.05). Compared with broiler chickens supplied with I_Se_ source, broiler chickens fed O_Se_ source decreased the ${\text{L}}_{24\text{h}}^{*}$, ${\text{L}}_{48\text{h}}^{*}$, drip loss, and shear force, and increased the ${\text{b}}_{24\text{h}}^{*}$ and ${\text{a}}_{48\text{h}}^{*}$ (*p* < 0.05). Broiler chickens supplied with H_EMB_ level increased the ${\text{b}}_{45\text{min}}^{*}$, ${\text{a}}_{24\text{h}}^{*}$, and ${\text{a}}_{48\text{h}}^{*}$ compared with the broiler chickens supplied with Z_EMB_ level (*p* < 0.05). In detail, the Y-Se + EM group had a greater ${\text{a}}_{24\text{h}}^{*}$ compared with the other groups, and S-Se + EM group had a greater ${\text{a}}_{24\text{h}}^{*}$ compared with the S-Se and Y-Se groups (*p* < 0.05). The Y-Se + EM group had a greater ${\text{b}}_{24\text{h}}^{*}$ compared with the other groups, and the S-Se group had a greater ${\text{b}}_{24\text{h}}^{*}$ compared with the S-Se + EM and Y-Se groups (*p* < 0.05). The Y-Se + EM group had a greater ${\text{a}}_{48\text{h}}^{*}$ compared with the other groups, and the S-Se group had a greater ${\text{a}}_{48\text{h}}^{*}$ compared with the S-Se + EM group (*p* < 0.05). The Y-Se + EM group had a greater ${\text{b}}_{48\text{h}}^{*}$ compared with the S-Se + EM and Y-Se groups (*p* < 0.05). The S-Se and S-Se + EM groups had a higher ${\text{L}}_{48\text{h}}^{*}$ compared with the Y-Se and Y-Se + EM groups (*p* < 0.05). The S-Se + EM group had a greater drip loss compared with the other groups (*p* < 0.05). The S-Se group had a greater shear force compared with the Y-Se and Y-Se + EM groups (*p* < 0.05).

### Muscle Fiber Characteristics in the Breast and Thigh Muscles

**Figure 1:**
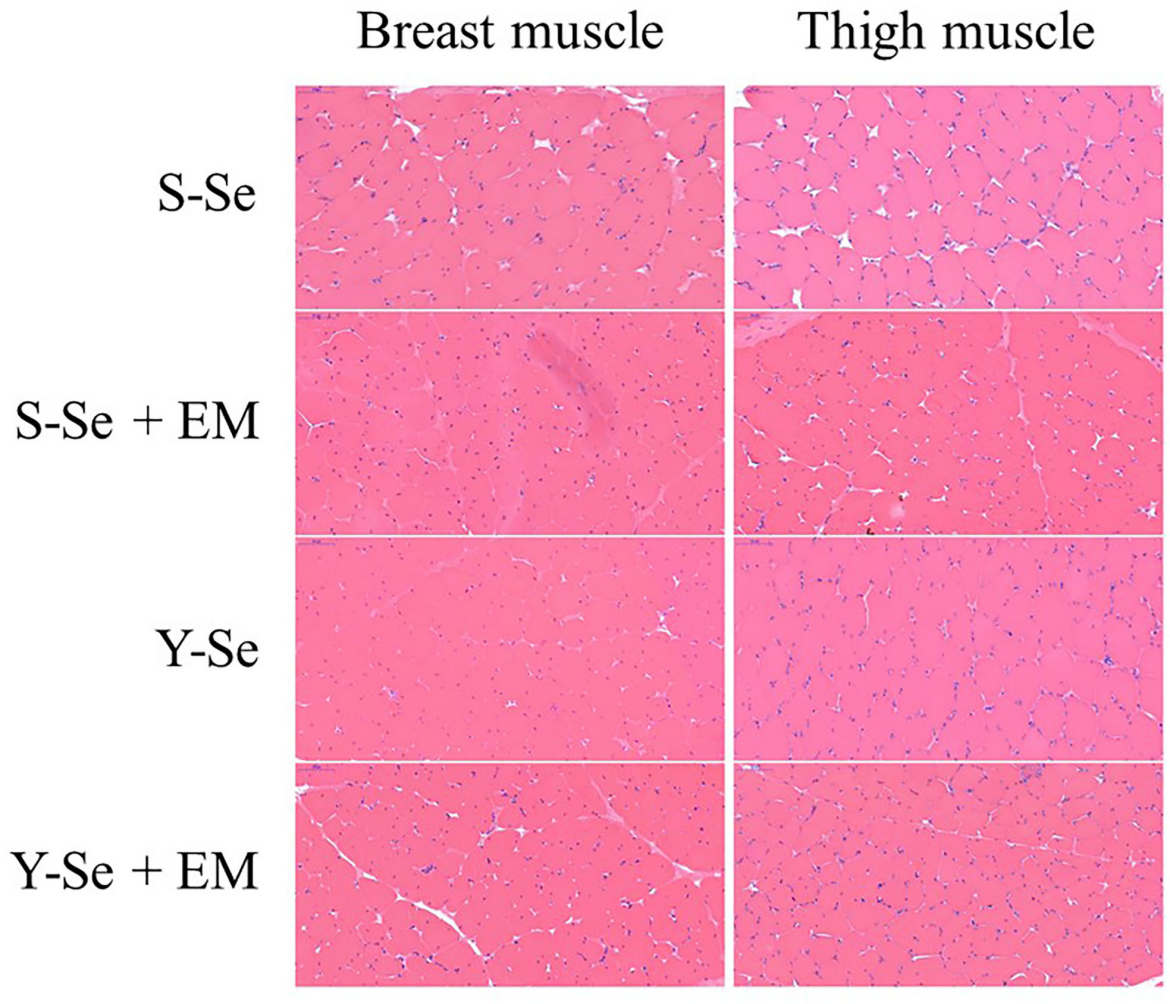
Hematoxylin and eosin staining of the right breast and thigh muscles (400 ×). S-Se, sodium selenite; S-Se + EM, sodium selenite + 0.5% EM; Y-Se, selenium yeast; Y-Se + EM, selenium yeast + 0.5% EM.

The effect of Se source and EM level on muscle fiber characteristics in the breast and thigh muscles is shown in Figure 1 and Table 6. In the breast muscle, there were no interactions between Se source and EM level regarding the muscle fiber characteristics (*p* > 0.05). Compared with the broiler chickens fed I_Se_ source, broiler chickens fed O_Se_ source decreased the perimeter, cross-sectional area, and diameter, and improved the density (*p* < 0.05). Broiler chickens supplied with H_EMB_ level decreased the cross-sectional area and diameter, and enhanced the density, compared with the broiler chickens supplied with Z_EMB_ level (*p* < 0.05). In detail, the S-Se group had a greater cross-sectional area and diameter compared with the other groups (*p* < 0.05). The Y-Se + EM group had a greater density compared with the S-Se and S-Se + EM groups, and the Y-Se group had a greater density compared with the S-Se group (*p* < 0.05).

**Table 6:** Effects of different Se sources and different bacteria supplementation levels on the muscle fiber characteristic of three-yellow chickens.

**Items**	**Groups**				**Main effect of S**		**Main effect of E**		**SEM**	***P-*** **value**			
	**S – Se**	**S – Se + EM**	**Y – Se**	**Y – Se + EM**	**I_**Se**_**	**O_**Se**_**	**Z_**EMB**_**	**H_**EMB**_**		**Treatment**	**S**	**E**	**S × E**
Breast muscle													
Perimeter (μm)	148.97	138.84	128.79	116.88	142.64^*^	123.69	137.44	130.61	4.75	0.146	0.033	0.230	0.920
Cross-sectional area (μm^2^)	1,694.13^a^	1,264.07^b^	1,197.35^b^	987.53^b^	1,479.10*	1,092.44	1,445.74^§^	1,125.80	89.08	0.008	0.006	0.015	0.317
Diameter (μm)	38.02^a^	31.71^b^	30.93^b^	29.45^b^	33.51*	29.95	34.47^§^	30.71	1.02	0.028	0.016	0.036	0.161
Density	75.61^c^	90.67^bc^	106.00^ab^	130.67^a^	85.02	116.57*	92.98	105.67^§^	6.29	0.011	0.002	0.047	0.600
Thigh muscle													
Perimeter (μm)	145.39	129.50	136.82	119.93	137.45	128.37	141.11^§^	124.71	3.93	0.061	0.113	0.022	0.916
Cross-sectional area (μm^2^)	1,691.05	1,280.20	1,315.96	1,087.79	1,485.63*	1,201.87	1,503.50^§^	1,184.00	90.84	0.053	0.047	0.033	0.413
Diameter (μm)	32.89	31.47	28.79	26.80	32.18*	27.59	30.84	28.67	0.99	0.056	0.018	0.238	0.833
Density	80.75^b^	91.89^b^	100.50^b^	140.89^a^	85.52	117.81*	90.63	116.39^§^	7.85	0.024	0.014	0.049	0.233

*S-Se, sodium selenite; S-Se + EM, sodium selenite + 0.5% EM; Y-Se, selenium yeast; Y-Se + EM, selenium yeast + 0.5% EM. S, Se source; E, EM level; S × E, interaction between Se source and EM level; I_Se_, inorganic Se; O_Se_, organic Se; H_EMB_, high EM; Z_EMB_, low EM.*

a−c *Values of group in the same row with the same superscript or absence of a superscript were not significantly different (p > 0.05).*

* *Values of the main effect of S in the same row were significantly different (p < 0.05).*

§ *Values of the main effect of E in the same row were significantly different (p < 0.05)*.

In the thigh muscle, there were no interactions between Se source and EM level regarding the muscle fiber characteristics (*p* > 0.05). Compared with the broiler chickens fed I_Se_ source, broiler chickens fed O_Se_ source decreased the cross-sectional area and diameter, and increased the density (*p* < 0.05). Broiler chickens supplied with H_EMB_ level decreased the perimeter and cross-sectional area, and increased the density, compared with the broiler chickens supplied with Z_EMB_ level (*p* < 0.05). In detail, the Y-Se + EM group had a greater density compared with the other groups (*p* < 0.05).

### The Correlations Between the Meat Quality and Muscle Fiber Characteristics

**Figure 2:**
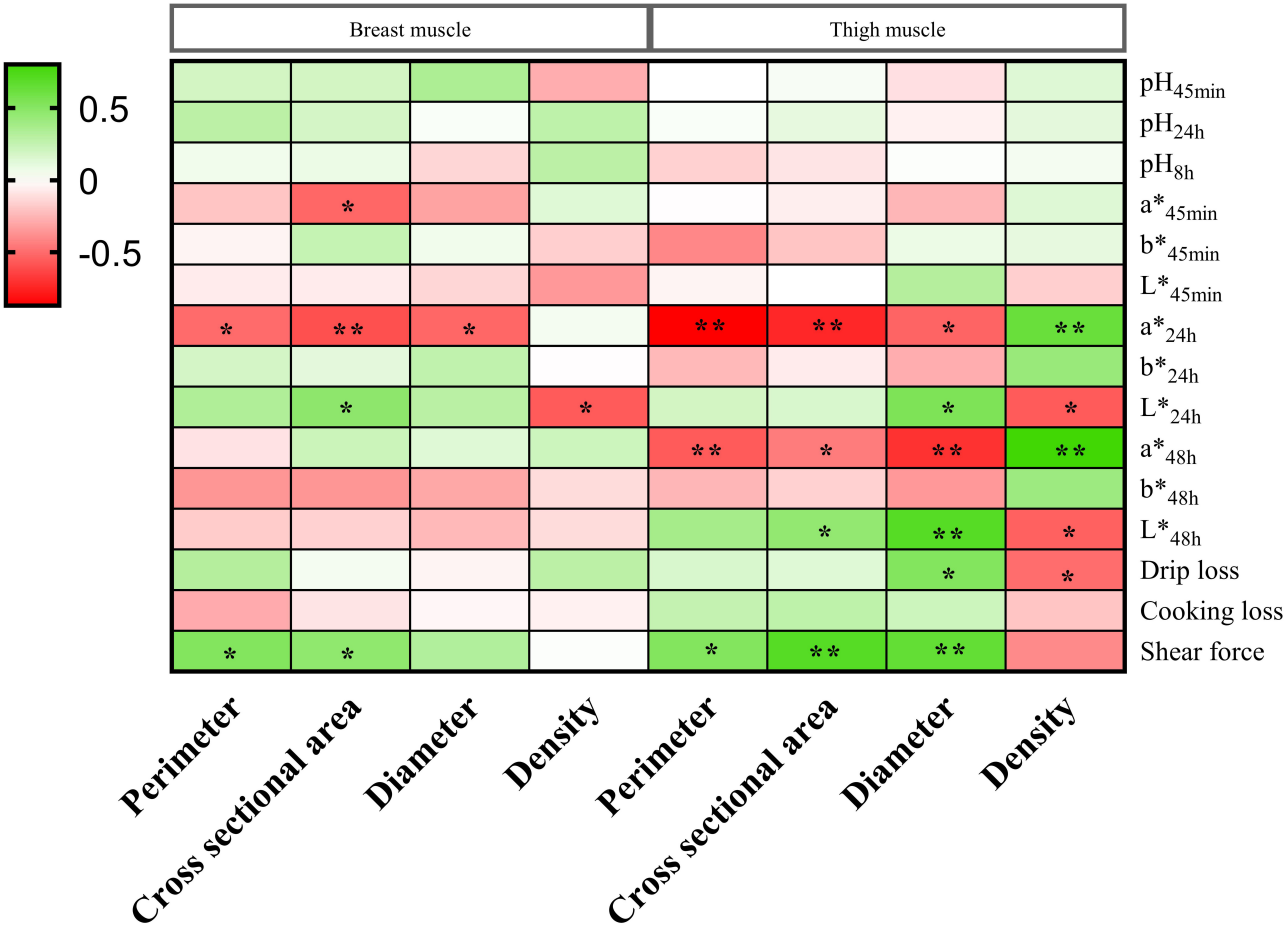
The correlations between the meat quality and muscle fiber characteristics in the breast and thigh muscles of three-yellow chickens. * means significant difference (*p* < 0.05); ** means highly significant difference (*p* < 0.01).

The correlations between the meat quality and muscle fiber characteristics in the breast and thigh muscles are shown in Figure 2. In the breast muscle, ${\text{a}}_{24\text{h}}^{*}$ showed a negative correlation with perimeter, but shear force showed a positive correlation with perimeter (*p* < 0.05). The ${\text{a}}_{45\text{min}}^{*}$ and ${\text{a}}_{24\text{h}}^{*}$ were negatively related to cross-sectional area, and ${\text{L}}_{24\text{h}}^{*}$ and shear force were positively related to cross-sectional area (*p* < 0.05). ${\text{a}}_{24\text{h}}^{*}$ showed a negative correlation with diameter (*p* < 0.05). ${\text{L}}_{24\text{h}}^{*}$ was negatively related to density (*p* < 0.05). In the thigh muscle, ${\text{a}}_{24\text{h}}^{*}$ and ${\text{a}}_{48\text{h}}^{*}$ showed negative correlations with perimeter, but shear force showed a positive correlation with perimeter (*p* < 0.05). ${\text{a}}_{24\text{h}}^{*}$ and ${\text{a}}_{48\text{h}}^{*}$ were negatively related to cross-sectional area; however, ${\text{L}}_{48\text{h}}^{*}$ and shear force were positively related to cross-sectional area (*p* < 0.05). ${\text{a}}_{24\text{h}}^{*}$ and ${\text{a}}_{48\text{h}}^{*}$ showed negative correlations with diameter, but ${\text{L}}_{24\text{h}}^{*}$, ${\text{L}}_{48\text{h}}^{*}$, drip loss, and shear force showed positive correlations with diameter (*p* < 0.05). ${\text{L}}_{24\text{h}}^{*}$, ${\text{L}}_{48\text{h}}^{*}$, and drip loss were negatively related to density, but ${\text{a}}_{24\text{h}}^{*}$ and ${\text{a}}_{48\text{h}}^{*}$ were positively related to density (*p* < 0.05).

### Expression Levels of Muscle Fiber-Relative Genes in the Breast and Thigh Muscles

The effect of Se source and EM level on the expression levels of muscle fiber-relative genes in the breast and thigh muscles is shown in Table 7. In the breast muscle, this study showed significant interactions between the Se source and EM level regarding the expression levels of *CAST* and *SM* (*p* < 0.05). Compared with the broiler chickens fed I_Se_ source, broiler chickens fed O_Se_ source decreased the expression level of *CAST* (*p* < 0.05) and increased the expression levels of *MRF4* and *CAPN2* (*p* < 0.05). Broiler chickens supplied with H_EMB_ level increased the expression levels of *MyoG* and *CAPN3* compared with the broiler chickens supplied with Z_EMB_ level (*p* < 0.05). In detail, the S-Se + EM and Y-Se + EM groups increased the expression level of *CAPN3* compared with the Y-Se group (*p* < 0.05). The S-Se group had a greater expression level of *CAST* compared with the other groups, and the S-Se + EM and Y-Se + EM groups had a greater expression level of *CAST* compared with the Y-Se group (*p* < 0.05).

**Table 7:** Effects of different Se sources and different bacteria levels on the expression levels of muscle fiber-relative genes in the breast and thigh muscles.

**Items**	**Groups**				**Main effect of S**		**Main effect of E**		**SEM**	***P-*** **value**			
	**S – Se**	**S – Se + EM**	**Y – Se**	**Y – Se + EM**	**I_**Se**_**	**O_**Se**_**	**Z_**EMB**_**	**H_**EMB**_**		**Treatment**	**S**	**E**	**S × E**
Breast muscle													
*Myf5*	1.01	0.66	0.92	0.88	0.79	0.90	0.96	0.76	0.07	0.317	0.639	0.179	0.273
*MyoG*	1.00	1.67	1.02	1.77	1.38	1.39	1.01	1.70^§^	0.15	0.135	0.823	0.030	0.877
*MRF4*	1.00	0.70	1.37	1.32	0.85	1.34*	1.15	1.01	0.12	0.152	0.045	0.414	0.546
*CAPN2*	1.03	1.15	1.45	1.57	1.09	1.51*	1.24	1.36	0.09	0.085	0.016	0.448	0.983
*CAPN3*	1.15^ab^	1.23^a^	0.73^b^	1.39^a^	1.18	1.02	0.94	1.31^§^	0.09	0.046	0.414	0.032	0.074
*CAST*	1.01^a^	0.78^b^	0.49^c^	0.82^b^	0.87*	0.68	0.69	0.81	0.06	0.000	0.001	0.240	0.000
*MSTN*	1.00	2.37	2.22	2.62	1.82	2.42	1.87	2.53	0.23	0.193	0.138	0.080	0.317
*MEF2A*	1.00	1.04	0.74	0.82	1.02	0.79	0.85	0.93	0.06	0.292	0.076	0.647	0.850
*MEF2D*	1.00	0.66	0.51	0.66	0.76	0.59	0.65	0.66	0.07	0.197	0.085	0.489	0.089
*FM*	1.00	1.06	0.89	0.80	1.03	0.85	0.95	0.93	0.06	0.436	0.166	0.875	0.514
*SM*	1.02	0.58	0.74	1.91	0.80	1.21	0.85	1.25	0.21	0.078	0.122	0.251	0.037
*IGF - I*	1.01	2.12	1.40	1.37	1.65	1.39	1.23	1.75	0.17	0.110	0.549	0.089	0.075
*IGF - II*	1.00	1.81	1.97	2.14	1.51	2.06	1.55	1.98	0.17	0.153	0.065	0.148	0.337
Thigh muscle													
*Myf5*	1.01^b^	3.14^a^	3.20^a^	4.20^a^	2.07	3.80*	1.88	3.67^§^	0.40	0.001	0.001	0.002	0.115
*MyoG*	1.00^b^	2.78^a^	0.76^b^	2.89^a^	1.71	1.82	0.86	2.85^§^	0.31	0.001	0.821	0.000	0.562
*MRF4*	1.02^ab^	1.41^a^	0.62^b^	1.43^a^	1.21	1.08	0.82	1.42^§^	0.11	0.005	0.188	0.001	0.141
*CAPN2*	1.00	1.39	1.22	2.32	1.23	1.66	1.13	1.76^§^	0.19	0.079	0.084	0.037	0.246
*CAPN3*	1.00^b^	0.57^b^	2.53^a^	2.42^a^	0.79	2.46*	1.61	1.50	0.32	0.023	0.004	0.527	0.704
*CAST*	1.03	0.50	0.70	1.29	0.77	1.05	0.90	0.90	0.13	0.071	0.269	0.863	0.021
*MSTN*	1.01	1.45	1.17	2.09	1.23	1.51	1.11	1.77	0.19	0.222	0.285	0.082	0.511
*MEF2A*	1.00^b^	1.31^b^	1.12^b^	4.08^a^	1.16	2.31*	1.07	2.70^§^	0.45	0.003	0.007	0.004	0.009
*MEF2D*	1.01^b^	0.94^b^	0.79^b^	1.85^a^	0.97	1.09	0.87	1.24^§^	0.13	0.031	0.121	0.033	0.018
*FM*	1.00^a^	0.83^a^	0.59^ab^	0.21^b^	0.92*	0.40	0.80	0.52	0.12	0.024	0.009	0.062	0.366
*SM*	1.07^b^	1.18^b^	2.93^a^	1.70^b^	1.12	2.31*	2.00	1.44	0.30	0.038	0.017	0.140	0.093
*IGF - I*	1.02^a^	0.43^bc^	0.28^c^	0.72^ab^	0.68	0.43	0.60	0.52	0.10	0.005	0.089	0.524	0.002
*IGF - II*	1.02^a^	0.38^b^	0.52^b^	1.31^a^	0.64	0.74	0.66	0.75	0.11	0.002	0.120	0.554	0.000

*S-Se, sodium selenite; S-Se + EM, sodium selenite + 0.5% EM; Y-Se, selenium yeast; Y-Se + EM, selenium yeast + 0.5% EM. S, Se source; E, EM level; S × E, interaction between Se source and EM level; I_Se_, inorganic Se; O_Se_, organic Se; H_EMB_, high EM; Z_EMB_, low EM; Myf5, myogenic factor 5; MyoG, myogenin; MRF4, myogenic regulatory factor 4; CAPN2, calpain 2; CAPN3, calpain 3; CAST, calpastatin; MSTN, myostatin; MEF2A, myocyte enhancer factor 2A; MEF2D, myocyte enhancer factor 2D; FM, fast myosin heavy chain; SM, slow myosin heavy chain; IGF- I, insulin-like growth factor I; IGF-II, insulin-like growth factor II; β-actin, beta-actin.*

a−c *Values of group in the same row with the same superscript or absence of a superscript were not significantly different (p > 0.05).*

* *Values of the main effect of S in the same row were significantly different (p < 0.05).*

§ *Values of the main effect of E in the same row were significantly different (p < 0.05)*.

In the thigh muscle, this study showed significant interactions between the Se source and EM level regarding the expression levels of *CAST, MEF2A, MEF2D, IGF-I*, and IGF-II (*p* < 0.05). Compared with the broiler chickens fed I_Se_ source, broiler chickens fed O_Se_ source decreased the expression level of *FM* and increased the expression levels of *Myf5, CAPN3, MEF2A*, and *SM* (*p* < 0.05). Broiler chickens supplied with H_EMB_ level increased the expression levels of *Myf5, MyoG, MRF4, CAPN2, MEF2A*, and *MEF2D* compared with the broiler chickens supplied with Z_EMB_ level (*p* < 0.05). In detail, the S-Se group had the lowest expression level of *Myf5* compared with the other groups (*p* < 0.05). The S-Se + EM and Y-Se + EM groups increased the expression level of *MyoG* compared with the S-Se and Y-Se groups (*p* < 0.05). The S-Se + EM and Y-Se + EM groups increased the expression level of *MRF4* compared with the Y-Se group (*p* < 0.05). The Y-Se and Y-Se + EM groups increased the expression level of *CAPN3* compared with the S-Se and S-Se + EM groups (*p* < 0.05). The Y-Se + EM group had the highest expression levels of *MEF2A* and *MEF2D* compared with the other groups (*p* < 0.05). The S-Se and S-Se + EM groups increased the expression level of *FM* compared with the Y-Se + EM group (*p* < 0.05). The Y-Se group had the highest expression level of *SM* compared with the other groups (*p* < 0.05). The S-Se group increased the expression level of *IGF-I* compared with the S-Se + EM and Y-Se groups, and the Y-Se + EM group increased the expression level of *IGF-I* compared with the Y-Se group (*p* < 0.05). The S-Se and Y-Se + EM groups increased the expression level of IGF-II compared with the S-Se + EM and Y-Se groups (*p* < 0.05).

## Discussion

Recently, the application of Y-Se and EM has received amounts of attention. The effect of dietary Y-Se supplementation on growth performance of chickens is controversial. Some studies show that dietary Y-Se supplementation increases the BW and weight gain, and decreases the FCR (5, 7). However, some studies show that dietary Y-Se supplementation has no influence on the BW, ADG, ADFI, and FCR of chicken during the overall period (28–30). Our results indicated that, compared with dietary I_Se_ source, dietary O_Se_ source only decreased the FCR during 1–35 days of three-yellow chickens. In this study, EM mainly included *Lactobacillus* and yeast. A previous study showed that dietary *Lactobacillus* increased the BW, ADG, and decreased the FCR (31). However, a previous study also showed that dietary *Lactobacillus* had no influence on the BW, ADG, ADFI, and increased the FCR (32). When supplied with 0.5% dried yeast, it decreases the ADFI of broiler chickens, but has no influence on the ADG and FCR (2). Our results indicated that compared with supplied with Z_EMB_ level, those supplied with H_EMB_ level had no influence on growth performance during the overall period. These discrepancies maybe related to the differences about the adding levels of bacteria and Se, bacteria species, and living environment.

Meat quality is an important aspect of the poultry industry and deeply influences the economic benefits. It can be reflected by the pH value, meat color, drip loss, cooking loss, and shear force. Meat colors include a^*^, b^*^, and L^*^, which is a major aspect to evaluate the products (33). Water-holding capacity, a key characteristic of meat quality, is reflected by drip loss and cooking loss (1). Shear force can reflect the tenderness of the meat (13), which is considered the most important index for eating quality (34). Previous studies had shown that dietary Y-Se supplementation decreased the drip loss and cooking loss of pigs and Ross 308 broilers (18, 22). Our results indicated that compared with dietary I_Se_ source, the broiler chickens fed O_Se_ source increased the meat quality by decreasing L^*^, drip loss, and shear force, and by increasing the a^*^ and b^*^ in the muscles of three-yellow chickens, which were in agreement with the previous studies. Dietary *Lactobacillus* supplement increases the b^*^ in the thigh muscle of AA broilers (10), and dietary yeast supplement decreases the shear force in drumstick of Ross broilers (13). Our results indicated that compared with supplied with Z_EMB_ level, broiler chickens supplied with H_EMB_ level improved the meat quality by increasing the pH, a^*^, and b^*^ in the muscles of three-yellow chickens. The mechanism of meat quality change may be related to the change in antioxidant levels. As an antioxidant mineral, Se is an essential co-factor in the antioxidant enzyme system (35). When supplied with Y-Se, *Lactobacillus* and yeast can increase the antioxidant capacity (2, 13, 18–20).

Our results showed that dietary O_Se_ source and H_EMB_ level decreased perimeter, cross-sectional area, and diameter, and increased density of muscle fiber on the breast and thigh muscles of three-yellow chickens. In the body of vertebrates, the most abundant tissue is skeletal muscle, and around 75–90% of skeletal muscle is composed of muscle fiber (36). Therefore, muscle fiber is a main factor to influence the meat quality. Muscle tenderness is negatively related to muscle fiber diameter and positively related to muscle fiber density (37). The glycogen concentration is low and the fat concentration is high in the slow muscle fiber (24), and it is positively related to pH and negatively related to drip loss and shear force (25, 38). However, the contents of myohemoglobin and chondriosome are low in the fast muscle fiber, which makes it easy to produce lactic acid (26), and it is positively related to drip loss, shear force, and L^*^, and negatively related to pH (24, 25). The present findings suggest that the muscle fiber characteristics were closely related to the meat quality, which were in agreement with the previous studies. Muscle fiber perimeter, cross-sectional area, and diameter were negatively related to a^*^ and were positively related to the shear force. Muscle fiber density was positively related to a^*^ and was negatively related to L^*^ and drip loss. Our study illuminated that O_Se_ source and H_EMB_ level could improve the meat quality by regulating the muscle fiber characteristics.

Myf5, MyoG, and MRF4 are the members of myogenic regulatory factor family (39). Myf5, as a myogenic determination factor, takes part in the specification and proliferation of myoblasts (40). MyoG and MRF4 lead myoblast to form multinucleated myofiber (41, 42). Our results showed that in the breast and/or thigh muscles of three-yellow chickens, O_Se_ source and/or H_EMB_ level increased the expression levels of Myf5, MyoG, and MRF4 genes. These results explained how Y-Se and EM increased the density of muscle fiber. Calpain belongs to the papain superfamily, which includes CAPN1, CAPN2, and CAPN3, and is a Ca^2+^-regulated proteolytic enzyme (43). It degrades the myofibrillar protein (44), which affects the diameter of muscle fiber. Postmortem proteolysis of myofibrillar protein deeply influences the tenderness of the meat (45). CAST specifically inhibits the activity of calpain (46). Our results showed that O_Se_ source and/or H_EMB_ level increased the expression levels of CAPN2 and CAPN3 genes in the breast and/or thigh muscles of three-yellow chickens, and O_Se_ source decreased the expression level of CAST gene in the breast muscle. This was a reason why Y-Se and EM decreased the perimeter, cross-sectional area, and diameter of muscle fiber, and it also explains how Y-Se decreased the shear force of muscles. MEF2A and MEF2D, as members of the MEF2 family, are DNA-binding transcription factors and play key roles in muscle development and differentiation (47). They are related to the formation of slow muscle fiber (48, 49). FM and SM are the fast myosin heavy chain and slow myosin heavy chain, respectively. Our results showed that in the thigh muscle O_Se_ source and H_EMB_ level increased the expression levels of MEF2A and MEF2D genes, and O_Se_ source increased the expression level of SM gene and decreased the expression level of FM gene. These results indicated that Y-Se and EM increased the number of slow muscle fibers, and Y-Se decreased the number of fast muscle fibers. This was a reason why Y-Se and EM decreased the perimeter, cross-sectional area, and diameter of muscle fiber, and it also explains how Y-Se and EM increased the meat quality.

## Data Availability Statement

The original contributions presented in the study are included in the article/supplementary materials, further inquiries can be directed to the corresponding author.

## Ethics Statement

The animal study was reviewed and approved by Committee of Laboratory Animal Management and Animal Welfare of Hunan Agricultural University.

## Author Contributions

JX: conceptualization and writing–original draft preparation. CF: methodology and resources. RM: validation and supervision. RZ: software and data curation. YX: visualization. YQ: formal analysis. JT: investigation. RF: writing–review and editing, project administration, and funding acquisition. All authors contributed to the article and approved the submitted version.

## Funding

This research was funded by the Double first-class construction project of Hunan Agricultural University and the National Key Research and Development Program of China, grant number 2018YFD0501403.

## Conflict of Interest

The authors declare that the research was conducted in the absence of any commercial or financial relationships that could be construed as a potential conflict of interest.

## Publisher's Note

All claims expressed in this article are solely those of the authors and do not necessarily represent those of their affiliated organizations, or those of the publisher, the editors and the reviewers. Any product that may be evaluated in this article, or claim that may be made by its manufacturer, is not guaranteed or endorsed by the publisher.
